# Supplementary material for: An examination of psychometric properties of study quality assessment scales in meta-analysis: Rasch measurement model applied to the firefighter cancer literature
Source: PLoS One. 2023 Jul 26;18(7):e0284469. doi: 10.1371/journal.pone.0284469 (PMC10370747; doi:10.1371/journal.pone.0284469)
Supplement: S1 Appendix — (DOCX) [file pone.0284469.s001.docx]

Appendix A. Included 49 studies

1. Ahn, Y. S., & Jeong, K. S. (2015). Mortality due to malignant and non-malignant diseases in Korean professional emergency responders. *PLoS One, 10*(3), 1-14. doi:10.1371/journal.pone.0120305
2. Ahn, Y. S., Jeong, K. S., & Kim, K. S. (2012). Cancer morbidity of professional emergency responders in Korea. *Am J Ind Med, 55*(9), 768-778. doi:10.1002/ajim.22068
3. Amadeo, B., Marchand, J.-L., Moisan, F., Donnadieu, S., Gaëlle, C., Simone, M.-P., . . . Brochard, P. (2015). French firefighter mortality: analysis over a 30-year period. *American Journal of Industrial Medicine, 58*(4), 437-443. doi:10.1002/ajim.22434
4. Aronson, K. J., Tomlinson, G. A. and Smith, L. (1994). Mortality among fire fighters in metropolitan Toronto. *American Journal of Industrial Medicine, 26*(1), 89-101. doi:10.1002/ajim.4700260108
5. Baris, D., Garrity, T. J., Telles, J. L., Heineman, E. F., Olshan, A., & Zahm, S. H. (2001). Cohort mortality study of Philadelphia firefighters. *American Journal of Industrial Medicine, 39*(5), 463-476. doi:<https://doi.org/10.1002/ajim.1040>
6. Bates, M. N., Fawcett, J., Garrett, N., Arnold, R., Pearce, N., & Woodward, A. (2001). Is testicular cancer an occupational disease of fire fighters? *American Journal of Industrial Medicine, 40*(3), 263-270. doi:10.1002/ajim.1097
7. Beaumont, J. J., Chu, G. S., Jones, J. R., Schenker, M. B., Singleton, J. A., Piantanida, L. G., & Reiterman, M. (1991). An epidemiologic study of cancer and other causes of mortality in San Francisco firefighters. *American Journal of Industrial Medicine, 19*(3), 357-372. doi:10.1002/ajim.4700190309
8. Daniels, R. D., Kubale, T. L., Yiin, J. H., Dahm, M. M., Hales, T. R., Baris, D., . . . Pinkerton, L. E. (2014). Mortality and cancer incidence in a pooled cohort of US firefighters from San Francisco, Chicago and Philadelphia (1950-2009). *Occup Environ Med, 71*(6), 388-397. doi:10.1136/oemed-2013-101662
9. Demers, P. A., Checkoway, H., Vaughan, T. L., Weiss, N. S., Heyer, N. J., & Rosenstock, L. (1994). Cancer incidence among firefighters in Seattle and Tacoma, Washington (United States). *Cancer Causes & Control, 5*(2), 129-135. doi:10.1007/BF01830258
10. Deschamps, S., Momas, I., & Festy, B. (1995). Mortality Amongst Paris Fire-Fighters. *European Journal of Epidemiology, 11*(6), 643-646. Retrieved from <https://www.jstor.org/stable/3582167>
11. Firth, H. M., Cooke, K. R., & Herbison, G. P. (1996). Male cancer incidence by occupation: New Zealand, 1972-1984. *International Journal of Epidemiology, 25*(1), 14-21. doi:10.1093/ije/25.1.14
    Glass, D. C., Del Monaco, A., Pircher, S., Vander Hoorn, S., & Sim, M. R. (2016). Mortality and cancer incidence at a fire training college. *Occupational Medicine, 66*(7), 536-542. doi:oi:10.1093/occmed/kqw
12. Glass, D. C., Del Monaco, A., Pircher, S., Vander Hoorn, S., & Sim, M. R. (2017). Mortality and cancer incidence among male volunteer Australian firefighters. *Occupational and Environmental Medicine, 74*(9), 628-638. doi:10.1136/oemed-2016-104088
13. Glass, D. C., Pircher, S., Del Monaco, A., Hoorn, S. V., & Sim, M. R. (2016). Mortality and cancer incidence in a cohort of male paid Australian firefighters. *Occup Environ Med, 73*(11), 761-771. doi:10.1136/oemed-2015-103467
14. Guidotti, T. L. (1993). Mortality of urban firefighters in Alberta, 1927-1987. *American Journal of Industrial Medicine, 23*(6), 921-940. doi:10.1002/ajim.4700230608
15. Hansen, E. S. (1990). A cohort study on the mortality of firefighters. *British Journal of Industrial Medicine, 47*(12), 805-809. doi:10.1136/oem.47.12.805
16. Ide, C. W. (2014). Cancer incidence and mortality in serving whole-time Scottish firefighters 1984-2005. *Occup Med (Lond), 64*(6), 421-427. doi:10.1093/occmed/kqu080
17. Kullberg, C., Andersson, T., Gustavsson, P., Selander, J., Tornling, G., Gustavsson, A., & Bigert, C. (2018). Cancer incidence in Stockholm firefighters 1958-2012: An updated cohort study. International Archives of Occupational and Environmental Health, 9(3), 285-291. doi:10.1007/s00420-017-1276-1
18. Lenahan, P., Gochfeld, M., Meng, Q., Robson, M., & Fagliano, J. (2018). *A 30-year study of cancer incidence in firefighters and police officers in New Jersey's four largest municipalities*. Dissertation. Graduate Program in Public Health. Rutgers, The State University of New Jersey. New Jersey.
19. Ma, F., Fleming, L. E., Lee, D. J., Trapido, E., & Gerace, T. A. (2006). Cancer incidence in Florida professional firefighters, 1981 to 1999. *Journal of Occupational and Environmental Medicine, 48*(9), 883-888. doi:10.1097/01.jom.0000235862.12518.04
20. Ma, F., Fleming, L. E., Lee, D. J., Trapido, E., Gerace, T. A., Lai, H., & Lai, S. (2005). Mortality in Florida professional firefighters, 1972 to 1999. *Am J Ind Med, 47*(6), 509-517. doi:10.1002/ajim.20160
21. Moir, W., Zeig-Owens, R., Daniels, R. D., Hall, C. B., Webber, M. P., Jaber, N., . . . Prezant, D. J. (2016). Post-9/11 cancer incidence in World Trade Center-exposed New York City firefighters as compared to a pooled cohort of firefighters from San Francisco, Chicago and Philadelphia (9/11/2001-2009). *American Journal of Industrial Medicine, 59*(9), 722-730. doi:10.1002/ajim.22635
22. Musk, A. W., Monson, R. R., Peters, J. M., & Peters, R. K. (1978). Mortality among Boston firefighters, 1915--1975. *Br J Ind Med, 35*(2), 104-108. doi:10.1136/oem.35.2.104
23. Petersen, K. K. U., Pedersen, J. E., Bonde, J. P., Ebbehoej, N. E., & Hansen, J. (2018). Long-term follow-up for cancer incidence in a cohort of Danish firefighters. *Occupational and Environmental Medicine, 75*(4), 263-269. doi:10.1136/oemed-2017-104660
24. Petersen, K. U., Pedersen, J. E., Bonde, J. P., Ebbehøj, N. E., & Hansen, J. (2018). Mortality in a cohort of Danish firefighters; 1970-2014. *Int Arch Occup Environ Health, 91*(6), 759-766. doi:10.1007/s00420-018-1323-6
25. Pukkala, E., Martinsen, J. I., Weiderpass, E., Kjaerheim, K., Lynge, E., Tryggvadottir, L., . . . Demers, P. A. (2014). Cancer incidence among firefighters: 45 years of follow-up in five Nordic countries. *Occup Environ Med, 71*(6), 398-404. doi:10.1136/oemed-2013-101803
26. Rosenstock, L., Demers, P., Heyer, N. J., & Barnhart, S. (1990). Respiratory mortality among firefighters. *British Journal of Industrial Medicine, 47*(7), 462-465. doi:10.1136/oem.47.7.462
27. Tornling, G. r., Gustavsson, P., & Hogstedt, C. (1994). Mortality and cancer incidence in stockholm fire fighters. *American Journal of Industrial Medicine, 25*(2), 219-228. doi:<https://doi.org/10.1002/ajim.4700250208>
28. Vena, J. E., & Fiedler, R. C. (1987). Mortality of a municipal-worker cohort: IV. Fire fighters. *American Journal of Industrial Medicine, 11*(6), 671-684. doi:10.1002/ajim.4700110608
29. Wende, K. E. (1996). *A study of mortality among city of Buffalo firefighters*. Graduate School of State University of New York at Buffalo.
30. Zeegers, M. P., Friesema, I. H., Goldbohm, R. A., & van den Brandt, P. A. (2004). A prospective study of occupation and prostate cancer risk. *Journal of Occupational and Environmental Medicine, 46*(3), 271-279. doi:10.1097/01.jom.0000116961.48464.6b
31. Zeig-Owens, R., Webber, M. P., Hall, C. B., Schwartz, T., Jaber, N., Weakley, J., . . . Prezant, D. J. (2011). Early assessment of cancer outcomes in New York City firefighters after the 9/11 attacks: an observational cohort study. *Lancet, 378*(9794), 898-905. doi:10.1016/S0140-6736(11)60989-6
32. Zeig-Owens, R., Webber, M. P., Hall, C. B., Schwartz, T., Jaber, N., Weakley, J., . . . Prezant, D. J. (2011). Early assessment of cancer outcomes in New York City firefighters after the 9/11 attacks: an observational cohort study. *Lancet, 378*(9794), 898-905. doi:10.1016/S0140-6736(11)60989-6
33. Bates, M. N., & Lane, L. (1995). Testicular cancer in fire fighters: a cluster investigation. *The New Zealand Medical Journal, 108*(1006), 334-337.
34. Berg, J. W., & Howell, M. A. (1975). Occupation and bowel cancer. *Journal of Toxicology and Environmental Health, 1*(1), 75-89. doi:10.1080/15287397509529309
35. Eliopulos, E., Armstrong, B. K., Spickett, J. T., & Heyworth, F. (1984). Mortality of firefighters in Western Australia. *British Journal of Industrial Medicine, 41*, 183-187. doi:10.1136/oem.41.2.183
36. Giles, G., Staples, M., & Berry, J. (1993). *Cancer incidence in Melbourne Metropolitan Fire Brigade members, 1980 - 1989*. Retrieved from Canada: Retrieved from <https://www.ncbi.nlm.nih.gov/pubmed/8334236>
37. Heyer, N., Weiss, N. S., Demers, P., & Rosenstock, L. (1990). Cohort mortality study of Seattle Fire Fighters: 1945 - 1983. *American Journal of Industrial Medicine, 17*, 493 -504. doi:10.1002/ajim.4700170407
38. Ide, C. W. (1998). Failing firefighters: A survey of causes of death and ill-health retirement in serving firefighters from 1985-94. *Occupational Medicine, 48*(6), 381-388.
39. Mastromatteo, E. (1959). Mortality in city firemen, II: A study of mortality in firemen of a city fire department. *A.M.A. Archives of Industrial Health, 20*, 227-233.
40. Demers, P., Martinsen, J. I., Weiderpass, E., Kjærheim, K., Lynge, E., Sparén, P., & Pukkala, E. (2011). Cancer incidence among Nordic firefighters. *Occupational and Environmental Medicine, 68*(Suppl 1), A19-A20. doi:10.1136/oemed-2011-100382.60
41. Glass, D. C., Del Monaco, A., Pricher, S., Vander Hoorn, S., & Sim, M. R. (2019). Mortality and cancer incidence among female Australian firefighters. *Occupational and Environmental Medicine, 0*, 1-7. doi:10.1136/oemed-2018-105336
42. Blair, A., Walrath, J., & Rogot, E. (Mortality patterns among U.S. veterans by occupation. I. Cancer). 1985. *Journal of the National Cancer Institute, 75*(6), 1039-1047.
43. Morton, W., & Marjanovic, D. (1984). Leukemia incidence by occupation in the Portland- Vancouver metropolitan area. *American Journal of Industrial Medicine, 6*(3), 185-205. doi:10.1002/ajim.4700060304
44. Alguacil J, P. M., Gustavsson P. (2003). Occupations with increased risk of pancreatic cancer in the Swedish population. *Occupational and Environmental Medicine*, 570–576. doi:10.1136/oem.60.8.570
45. Guenel, P., Engholm, G., & Lynge, E. (1990). Laryngeal cancer in Denmark: a nationwide longitudinal study based on register linkage data. *Br J Ind Med, 47*(7), 473-479. doi:10.1136/oem.47.7.473
46. Dolin, P. J., & Cook-Mozaffari, P. (1992). Occupation and bladder cancer: a death-certificate study. *British Journal of Cancer, 66*(3), 568-578. doi:10.1038/bjc.1992.316
47. Pion, I. A., Rigel, D. S., Garfinkel, L., Silverman, M. K., & Kopf, A. W. (1995). Occupation and the risk of malignant melanoma. *CANCER Supplement, 75*(2), 637-644. doi:10.1002/1097-0142(19950115)75:2+<637::aid-cncr2820751404>3.0.co;2-#
48. McDowall, M. E., & Balarajan, R. (1986). Testicular cancer mortality in England and Wales 1971-80: variations by occupation. *Journal of Epidemiology and Community Health, 40*, 26-29. doi:10.1136/jech.40.1.26
49. Band, P. R., Le, N. D., Fang, R., & Gallagher, R. (2004). Identification of occupational cancer risks in British Columbia: a population-based case-control study of 769 cases of non-Hodgkin's lymphoma analyzed by histopathology subtypes. *Journal of occupational and environmental medicine*, *46*(5), 479–489. <https://doi.org/10.1097/01.jom.0000126028.99599.36>

Bottom of Form
