# Supplementary material for: An examination of psychometric properties of study quality assessment scales in meta-analysis: Rasch measurement model applied to the firefighter cancer literature
Source: PLoS One. 2023 Jul 26;18(7):e0284469. doi: 10.1371/journal.pone.0284469 (PMC10370747; doi:10.1371/journal.pone.0284469)
Supplement: Attachment — Submitted filename: PLOS ONE RR_Letter_responses.docx [file pone.0284469.s002.docx]

Dear Editor and Reviewers:

Thank you for the great feedbacks on our paper, which have improved our paper tremendously. Please find our responses on each comment made by editors and reviewers below. We are looking forward to publishing our paper in PLOS ONE.

Thank you so much.

| Comments made by editors and reviewers | Responses | Page #/ Line # |
| --- | --- | --- |
| Please ensure that your manuscript meets PLOS ONE's style requirements, including those for file naming. The PLOS ONE style templates can be found at  [https://journals.plos.org/plosone/s/file?id=wjVg/PLOSOne_formatting_sample_main_body.pdf](https://nam10.safelinks.protection.outlook.com/?url=https%3A%2F%2Fjournals.plos.org%2Fplosone%2Fs%2Ffile%3Fid%3DwjVg%2FPLOSOne_formatting_sample_main_body.pdf&data=05%7C01%7Cs.ahn%40miami.edu%7Cf8815f1e55414677a64c08db16a86faa%7C2a144b72f23942d48c0e6f0f17c48e33%7C0%7C0%7C638128684901032617%7CUnknown%7CTWFpbGZsb3d8eyJWIjoiMC4wLjAwMDAiLCJQIjoiV2luMzIiLCJBTiI6Ik1haWwiLCJXVCI6Mn0%3D%7C3000%7C%7C%7C&sdata=Y3JggbGTuu6VaNUboO0gCVVDdsY8PRkQol%2BfB7p%2F1X4%3D&reserved=0) and  [https://journals.plos.org/plosone/s/file?id=ba62/PLOSOne_formatting_sample_title_authors_affiliations.pdf](https://nam10.safelinks.protection.outlook.com/?url=https%3A%2F%2Fjournals.plos.org%2Fplosone%2Fs%2Ffile%3Fid%3Dba62%2FPLOSOne_formatting_sample_title_authors_affiliations.pdf&data=05%7C01%7Cs.ahn%40miami.edu%7Cf8815f1e55414677a64c08db16a86faa%7C2a144b72f23942d48c0e6f0f17c48e33%7C0%7C0%7C638128684901032617%7CUnknown%7CTWFpbGZsb3d8eyJWIjoiMC4wLjAwMDAiLCJQIjoiV2luMzIiLCJBTiI6Ik1haWwiLCJXVCI6Mn0%3D%7C3000%7C%7C%7C&sdata=BwF%2BaTv1PlKxY3jkW4vpK8JRNkawbbHQysitQ10TWMo%3D&reserved=0) | Manuscript have been formatted based on PLOS ONE’s style. | NA |
| a) Please clarify the sources of funding (financial or material support) for your study. List the grants or organizations that supported your study, including funding received from your institution.  b) State what role the funders took in the study. If the funders had no role in your study, please state: “The funders had no role in study design, data collection and analysis, decision to publish, or preparation of the manuscript.” c) If any authors received a salary from any of your funders, please state which authors and which funders. d) If you did not receive any funding for this study, please state: “The authors received no specific funding for this work.”  Please include your amended statements within your cover letter; we will change the online submission form on your behalf. | We included this information in our cover letter and highlighted in yellow. | pg. 1 in cover letter |
| Please review your reference list to ensure that it is complete and correct. If you have cited papers that have been retracted, please include the rationale for doing so in the manuscript text, or remove these references and replace them with relevant current references. Any changes to the reference list should be mentioned in the rebuttal letter that accompanies your revised manuscript. If you need to cite a retracted article, indicate the article’s retracted status in the References list and also include a citation and full reference for the retraction notice. | Completed. | NA |
| The NOQAS, by design, is meant to be adapted to different topics, so is functionally not the same scale for different users. The authors adapted it to their topic (firefighters/cancer), which was appropriate. However, I think this aspect should be discussed when comparing psychometric properties of this tool. | Thank you. This is a great point that we added into our discussion. | Line 369-373 |
| I think it would be helpful to add a sentence describing the training/characteristics of coders for this systematic review – this will help in understanding how easy/hard it was for them to apply these quality assessment tools. | Thank you. These were added to our method section. | Line # 225-230 |
| The authors note that of 49 studies included in their review, only 47 were able to be evaluated by raters using NOQAS (page 7 in the second start of page numbering). Why? | We made a simple mistake, but it has been corrected in the current document. | Line #346 |
| Can the Rasch measurement model be cited when first mentioned in the introduction? (Page 3) | Thank you. As suggested, the Rasch measurement model was cited on page 3. | Line #77 |
| PsycINFO does not have an H in it. (Page 6) | Thank you. It is corrected. |  |
| Need to add a (1) before standardized incidence ratio. (Page 10 if the page numbering were consistent) | Thank you. They are corrected. |  |
| Table 7 is a bit long and may be more helpful as supplemental material. | Although it is long, we would like to include Table 7 in the text. It is because we would like to show how each study and scale fit each other. | NA |
| Discussion: Need to add citation to Margulis and her colleague(s) – page 11 in the new numbering. | Thank you, We added citation. | Line # 406 |
| Some reference formatting looks like it got off (perhaps with reference management software) so should be double-checked prior to publication. | Checked and completed. | NA |
| Please add the more latest citation in the paper. | We believe that our literature review has been exhaustive in including the latest relevant literature for the topic. However, if you have any suggestions for literature we may have missed, please let us know. | NA |
| Compared the discussion with latest exiting literature. | We believe that our literature review has been exhaustive in including the latest relevant literature for the topic. However, if you have any suggestions for literature we may have missed, please let us know. | NA |
| In Detailed, Please explained the Research gap. | We added a bit more details in the introduction. However, we believe that our section – Study quality assessments in observational studies – already has included the research gaps found in our literature review. | Line # 67-69 |
| Please Add the research hypothesis and Research Question. | We added research questions on page 4 | Line #81-83 |
| Check the style of the reference according to the requirement of the journal. | Checked. |  |
